# Diaphragmatic Rupture and Hernia after Cardiopulmonary Resuscitation

**Published:** 2017

**Authors:** Feridoun Sabzi, Reza Faraji

**Affiliations:** Preventive Cardiovascular Research Center, Kermanshah University of Medical Sciences, Kermanshah, Iran

**Keywords:** Diaphragmatic hernia, Double valve replacement, Cardiopulmonary resuscitation

## Abstract

A 55-year-old man underwent surgical replacement of a mitral valve 10 years earlier. In a retrospective evaluation of a chest radiograph, the diaphragm was intact at the time of initial surgery. He was then admitted to our emergency room with a complaint of vertigo. During evaluation, he developed decreased consciousness. Ventricular fibrillation was diagnosed, and external massage and full cardiopulmonary resuscitation were performed. After 20 minutes, his sinus rhythm returned and hemodynamic status stabilized with inotropic drugs. Transthoracic echocardiography showed normal valvular function and no evidence of left cardiac malfunction or clot. Electrocardiography showed ST elevation in inferior leads, and levels of cardiac enzymes were elevated. Angiography showed an embolic lesion in the mid right coronary artery that was treated with percutaneous coronary intervention (PCI) and insertion of a stent. After 24 hours, the patient was extubated in good condition, but had mild dyspnea that progressed to CO_2_ narcosis and subsequent reintubation. Post-extubation chest radiography showed herniation of abdominal organs into the right hemithorax. The diaphragmatic defect was closed with a polytetrafluoroethylene patch by a thoracic surgeon, and the postoperative course was uncomplicated.

## INTRODUCTION

Damage to abdominal organs is a rare but known complication of cardiopulmonary resuscitation (CPR), usually with serious consequences. Injury of the liver, spleen, and stomach has been reported (1). We performed a PubMed search from 1970 to 2015 and found four reported cases of liver injury and three of gastric perforation following CPR; no case of diaphragmatic rupture and herniation was reported (2,3). The possibility of abdominal organ and diaphragmatic injury should be considered in cases of vigorous CPR. In this case, an embolic right coronary artery (RCA) lesion was the cause of cardiac arrest. Prolonged CPR was associated with diaphragmatic damage, which was only discovered when the patient had respiratory distress, enabling appropriate diagnostic imaging and therapy (4). Diaphragmatic hernia is a rare consequence of gastroepiploic artery (GEA) graft harvesting, as the route is constructed by making a canal through the diaphragm. The clinical signs and symptoms usually include epigastric pain, vomiting, or a combination of these symptoms. In contrast to diaphragmatic hernia development following coronary artery bypass grafting (CABG) with use of the GEA, a diaphragmatic hernia following CPR usually presents with respiratory distress related to the site of the defect and involves adjacent abdominal organs (5). Herniation of abdominal organs may cause strangulation and ulceration, and may be associated with respiratory difficulty. Multiple predisposing factors can lead to the development of a hernia through the normal diaphragmatic orifice, in addition to being induced by rupture of the diaphragm, or following creation of a tunnel for GEA graft passage (6). This complication can occur in the presence of a high body mass index; a sustained increase in intraabdominal pressure can lead to gradual iatrogenic enlargement of the diaphragmatic orifice or conduit passage route, and subsequent hernia formation (7).

## CASE SUMMARIES

**Figure 1.**
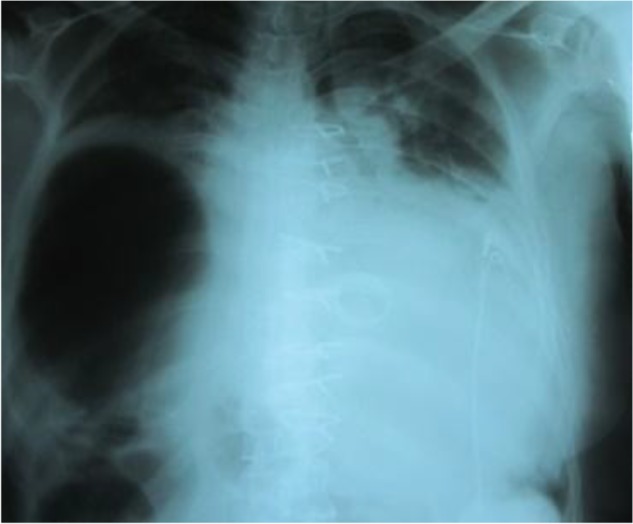
Herniation of abdominal organs into right hemithorax

A 55-year-old man with a history of mitral valve replacement 10 years prior presented to our center, complaining of respiratory distress and diaphoresis. At the time of the primary surgery, both pleural spaces were intact, and no damage to the phrenic nerve or diaphragm was reported. The postoperative course was unremarkable, and the patient was extubated the next morning, with mild residual respiratory distress. The post-extubation chest radiograph showed no evidence of intrathoracic herniation of abdominal organs. The patient was then readmitted with decreased consciousness and chest pain. While awaiting monitoring and review of vital signs, his level of consciousness further decreased and his blood pressure decreased to 65/45 mmHg. This was managed vigorously with 500 mL of crystalloid plus 500 mL of 5% dextrose. These temporarily stabilized his hemodynamic condition, and his blood pressure recovered to 80/50 mmHg. However, monitored ventricular tachycardia developed and was treated with external shock. The ventricular tachycardia converted to ventricular fibrillation; CPR was started and external shock was repeated. After recovery of a normal sinus rhythm, the blood pressure was maintained with inotropic drugs. The patient was examined by a senior cardiologist who made a diagnosis of acute inferior myocardial infarction; an order was then given to proceed to the angiography room with resuscitation in progress. The patient was intubated by the anesthesia nurse, and was transferred directly for PCI. Angiography showed an embolic lesion in the mid RCA. The lesion was severely stenotic and was treated by insertion of a drug-eluting stent. After extubation the next morning, the patient complained of abdominal pain and mild respiratory distress. The abdominal symptoms were evaluated by a general surgeon, and an acute abdominal problem was ruled out. The duration of CPR was 20 minutes, during which pulseless electrical activity was present. An intensive care unit nurse and an anesthesia nurse, both of whom were well-trained in CPR, performed sternal compression. The total time taken to restore cardiac output was approximately 35 minutes. The next morning, a chest radiograph revealed herniation of abdominal organs into the right hemithorax but rib fractures were not observed on three subsequent daily X-rays (Figure 1). The patient was extubated the next morning and had persistent mild respiratory distress, which increased progressively. Due to reduced consciousness, reintubation was performed. He was recommended thoracic surgery to repair the diaphragmatic hernia. During upper midline laparotomy, the abdomen was explored and revealed a large diaphragmatic hole measuring 10 × 12 cm. The defect was fresh and did not have a fibrotic edge or an adherent colon. The herniated colon was easily returned into the abdomen via the diaphragmatic defect. The hepatic surface was dissected free of the diaphragm, and the diaphragmatic hole was repaired with a 15×15 cm polytetrafluoroethylene synthetic surgical patch. The patient recovered uneventfully, and was discharged on the 8^th^ postoperative day.

## DISCUSSION

There are three important causes of diaphragmatic hernia following cardiac surgery. A patient with an undiagnosed congenital diaphragmatic hernia may have undergone cardiac surgery in adulthood. In a congenital diaphragmatic hernia, the defect is usually found in the left hemithorax; in most cases, there is no definitive sac. This type of hernia usually presents early after birth with respiratory difficulty, while the severity of dyspnea is related to the size and content of a herniated abdominal sac protruding into the hemithorax. Rarely, such a hernia remains undetected until late childhood, or it may be detected in adults during surgical evaluation when a patient develops severe dyspnea. In a second type, GEA harvesting is a causative factor. In the two decades since the first use of the right GEA, rare cases of diaphragmatic hernia have been reported after CABG (8). The clinical signs and symptoms in this type of hernia usually include epigastric pain, vomiting, or a combination of these symptoms. In contrast with hernia development following CABG with use of the GEA, diaphragmatic hernia detected following CPR usually presents with respiratory distress related to the site of the defect and affects adjacent abdominal organs. Herniation of the abdominal organs may cause strangulation and ulceration, and may be associated with respiratory difficulty. Multiple predisposing factors can lead to the development of a hernia through the normal diaphragmatic orifice, in addition to being induced by rupture of the diaphragm, or following creation of a tunnel for GEA graft passage. In the presence of a cough or obesity, a large hole created for passage of the GEA graft will most likely lead to a diaphragmatic hernia (9). Other causes unrelated to cardiac surgery include blunt abdominal trauma and crush injury; however, CPR as a factor in diaphragmatic rupture had not been previously reported. In this third type, diaphragmatic hernia occurred after CPR. Herein, we report a case of intrathoracic diaphragmatic hernia, which occurred 10 years after initial valve surgery, following vigorous CPR (10–12).
